# The Role of Large Language Models in Ophthalmology: A Review of Current Applications, Performance, and Future Directions

**DOI:** 10.7759/cureus.97374

**Published:** 2025-11-20

**Authors:** Liora Cohen, Anya R Gupta, Prisha Patel, Gurnoor S Gill, Harnaina Bains, Shailesh Gupta

**Affiliations:** 1 Clinical Trials and Technology, Advanced Research LLC, Deerfield Beach, USA; 2 Science, Florida Atlantic University High School, Boca Raton, USA; 3 Science, American Heritage School, Plantation, USA; 4 Medicine, Florida Atlantic University Charles E. Schmidt College of Medicine, Boca Raton, USA; 5 Ophthalmology, Broward Health North, Deerfield Beach, USA

**Keywords:** ai in ophthalmology, artificial intelligence in medicine, large language models (llm), medical education, ophthalmology review

## Abstract

Large language models (LLMs) are having a significant effect on clinical care as they become increasingly common in society. LLMs are artificial intelligence (AI) systems trained on a large amount of data that use deep learning to understand and generate human language. This enables these models to perform tasks such as answering questions, retrieving necessary information, and summarizing lengthy texts. Ophthalmology represents a unique field where numerous opportunities and innovations can be applied through these models. This review gathers recent studies to assess the current use of LLMs, such as Chat Generative Pretrained Transformer (ChatGPT), in ophthalmic areas, including clinical decision support, documentation, patient education, and medical training. LLMs have shown a remarkable capacity in diagnostic tasks, with GPT-4's performance on ophthalmology board-style assessments and subspecialty diagnostic reasoning matching or almost matching human-level performance. LLMs can also greatly increase workflow efficiency by automating note-taking and patient communication. They can enhance the efficacy and accessibility of medical education by creating educational materials and practice resources. By merging visual inputs with domain-specific data, multimodal and retrieval-augmented models improved their capacity to provide information with greater accuracy and relevance. These technologies are especially helpful in image-rich disciplines, such as ophthalmology, as they can assist in bridging the gap between visual diagnosis and text-based reasoning. Despite its benefits, incorporating AI into clinical care also has serious drawbacks, including the potential for ethical and legal dilemmas, as well as variations in the performance of specialists. LLM trends suggest that advanced, adaptable technologies that maintain anonymity hold considerable promise for enhancing clinical decision-making.

## Introduction and background

Artificial intelligence (AI) is increasingly influencing medical education, diagnostic processes, and healthcare delivery. Among the most widely adopted AI applications are large language models (LLMs), which are deep learning algorithms trained on vast corpora of online text, books, scientific literature, and medical documents. These systems can understand, analyze, and generate human-like text, enabling tasks such as answering clinical questions, drafting clinical documentation, supporting patient education, and simulating diagnostic reasoning [1,2].

Ophthalmology, a field that is both visually intensive and documentation-heavy, is particularly well-suited for the integration of LLMs. Routine ophthalmic practice involves detailed narrative documentation, communication of complex findings, and a high volume of patient interactions, all of which lend themselves to language-based automation. LLMs have demonstrated potential in supporting clinical narratives, assisting with specialty diagnosis, producing referral letters, and simplifying complex medical information for patients [1-5]. They are also becoming increasingly valuable in medical education, with studies showing that they can generate board-style questions and distill complex ophthalmic material into accessible learning tools for trainees [4,6].

Despite these advantages, the application of LLMs to ophthalmology remains constrained by significant challenges. These include concerns regarding patient data privacy, medicolegal accountability, and acceptance among both physicians and patients [3,7]. Furthermore, current models are not yet capable of fully interpreting ophthalmic images, and they remain prone to producing "hallucinations," outputs that appear fluent but are clinically inaccurate [8,9]. Such limitations underscore the need for oversight and careful evaluation prior to widespread clinical adoption.

The purpose of this review is to synthesize the current state of LLM applications in ophthalmology, including their diagnostic utility, clinical decision support, educational roles, and contributions to patient communication. In addition, this review will highlight logistical, ethical, and regulatory considerations while outlining opportunities for future development, particularly as LLMs evolve toward multimodal (models that can integrate and analyze multiple types of input, such as text and medical images) and domain-specific systems [10-14]. By clarifying both the potential and limitations of these technologies, this article aims to provide clinicians, educators, and researchers with a balanced framework for understanding and responsibly applying LLMs in ophthalmology.

## Review

Methods

This structured narrative review included peer-reviewed articles and preprints published in English between January 2020 and April 2024 that discussed or evaluated the application of LLMs in ophthalmology. Eligible studies focused on general-purpose or domain-specific LLMs, such as GPT-3.5, GPT-4, Bard, the Pathways Language Model (PaLM), Med-PaLM, ChatDoctor, and retrieval-augmented ophthalmology-specific models. To be considered for inclusion, studies had to demonstrate relevance to ophthalmic practice and address one or more of the following themes: clinical applications (e.g., diagnosis, decision support, or workflow optimization), documentation and communication tools, patient education, educational utility, or ethical, regulatory, or legal considerations specific to ophthalmology. Both experimental studies, such as those benchmarking LLMs on diagnostic or board-style tasks, and structured reviews were included. We excluded articles not published in English, studies published prior to 2020, and publications that focused exclusively on non-ophthalmic specialties or on imaging-only AI systems without an LLM component. Editorials, opinion pieces, media reports lacking original data, and articles without a structured analysis of ophthalmology-related LLM applications were also excluded, providing the final article count [10-56].

A targeted literature search was conducted between March and April 2024 using PubMed and Semantic Scholar. Search terms included combinations of "large language models", "LLMs", "GPT", "generative AI", "ophthalmology", "clinical applications", "education", and "ethics". In addition to database searches, we screened the reference lists of relevant articles to identify additional eligible publications. Titles and abstracts were reviewed for relevance, followed by full-text screening based on the predetermined inclusion and exclusion criteria. For each included study, we extracted information related to publication year, model type, study design, and application domain within ophthalmology. Given the narrative scope of this review, no formal risk-of-bias assessment was performed. Furthermore, no original statistical modeling or meta-analysis was performed; all quantitative results (e.g., P-values, confidence intervals, and pooled estimates) are directly reported from the original studies or existing published meta-analyses.

Clinical applications

Patient Triage and Diagnosis Support 

In ophthalmology, LLMs have increasingly supported clinicians' diagnostic reasoning and structured interpretation of patient data [1]. When tested on ophthalmic clinical scenarios, GPT-4 has demonstrated strong diagnostic capability relative to earlier models and human comparators on board-style assessments [2]. These findings suggest that LLMs can approximate expert-level reasoning when provided with structured clinical histories and examination findings [3]. In prospective and case-based evaluations, GPT-4 demonstrated diagnostic performance that approached that of board-certified specialists in neuro-ophthalmic disorders [4]. Benchmark comparisons across subspecialties provide additional insight beyond raw scores, highlighting strengths and weaknesses that vary by domain [5]. LLMs tend to perform better on text-rich tasks (e.g., general and neuro-ophthalmology scenarios) where narrative clues predominate [6]. Conversely, performance declines when substantial image interpretation is required (e.g., retinal or corneal imaging) unless multimodal inputs are incorporated [7]. These subspecialty benchmarks help delineate where LLMs are most useful and where caution is warranted due to modality and confidentiality constraints [8]. LLMs can also propose reasonable alternative diagnoses, supporting broader differential generation in complex or rare presentations [9]. Such tools may aid triage and initial decision-making in telemedicine or resource-limited settings where specialist access is constrained [10]

In Cai et al. [2], GPT-4 achieved an accuracy rate of over 80% on ophthalmology board-style questions, significantly outperforming GPT-3.5 and approaching the mean human results, while Wu et al. [5] confirmed these findings through a meta-analysis across multiple datasets. Beyond static question sets, prospective case-based evaluations demonstrate that GPT-4 can synthesize historical information, symptoms, and examination notes to propose accurate preliminary diagnoses [4,9]. Madadi et al. [4] demonstrated diagnostic agreement rates exceeding 85% for neuro-ophthalmic disorders, suggesting that LLMs can complement clinician reasoning where narrative detail predominates. However, accuracy declines for image-intensive conditions such as retinal vascular occlusions, keratitis, or uveitis, where multimodal inputs are critical [7].

LLMs also facilitate automated triage by classifying clinical presentations according to urgency. Lyons et al. [10] showed that GPT-4 achieved comparable triage accuracy to trained ophthalmic residents for differentiating emergent versus routine conditions. Similarly, Zandi et al. [8] found that GPT-4's triage recommendations for common complaints (e.g., red eye, vision loss, floaters) aligned with human ophthalmologists in over 90% of cases. These applications may improve tele-ophthalmology workflows by providing structured triage prior to referral, especially valuable in resource-limited or remote settings where ophthalmic specialists are unavailable.

Clinical Documentation and Workflow Optimization 

LLMs can reduce the administrative burden of ophthalmic care by assisting with documentation and communication tasks [11]. In practice, GPT-based systems can draft after-visit summaries, referral letters, and postoperative instructions aligned to clinic protocols [12]. Early evaluations of ambient AI scribe technology show associations with improved documentation efficiency and reduced perceived burden among clinicians [13]. In a cohort study evaluating the Nuance DAX ambient scribe, clinicians reported 30-40% reductions in documentation time and significant improvements in satisfaction and chart accuracy [11,12].

In ophthalmology, documentation is heavily narrative, including operative notes, slit-lamp findings, and follow-up recommendations, tasks that are highly amenable to structured LLM assistance [15,16]. Integration of LLMs within electronic health records (EHRs) can auto-populate ICD codes, identify missing elements, and generate structured summaries for insurance and billing [15,16]. Heilmeyer et al. [17] demonstrated the viability of open-source local LLMs for secure note generation within hospital firewalls, preserving Health Insurance Portability and Accountability Act (HIPAA) compliance.

Moreover, ambient listening technologies combined with ophthalmic dictation can capture examination details in real-time, reducing transcription errors and improving continuity of care [11,12,16]. Bongurala et al. [18] highlighted improved clinical safety and reduced chart discrepancies when AI-generated drafts were human-verified before finalization. Such improvements may mitigate medicolegal risks associated with delayed or incomplete charting while freeing ophthalmologists to focus on direct patient interaction.

Beyond drafting notes, LLM-enabled workflows can standardize documentation and improve clarity in settings with language barriers [14]. Integrations with EHRs are being explored to assist with code assignment, metric reporting, and the identification of missing clinical elements [15]. These capabilities may also improve billing accuracy while mitigating time pressures that contribute to burnout [16].

Enabling near-real-time documentation during visits can shorten the lag between encounter and chart completion [17]. Simultaneous, AI-assisted note generation may improve fidelity of the clinical record when reviewed and finalized by the clinician [18]. Such improvements could attenuate medicolegal risk associated with delayed or incomplete documentation when paired with human oversight [19]. Pilot work further supports the feasibility of localized, privacy-preserving LLMs for clinical note generation within health systems [20].

Patient Education and Communication

Applications of LLMs for patient-facing education are rapidly expanding in ophthalmology [21]. Head-to-head studies show that GPT-4 produces more understandable cataract-surgery education materials than Bard, as measured by the Patient Education Materials Assessment Tool for Printable Materials (PEMAT-P) criteria [22]. Azzopardi et al. found that ChatGPT-4 produced cataract-surgery information sheets that scored significantly higher on the PEMAT-P than Bard [21]. Similarly, Riazi Esfahani et al. reported that ChatGPT responses to common ophthalmology questions were more accurate, readable, and patient-appropriate than pre-vetted web materials [22].

Beyond static content, LLM chatbots embedded in patient portals or tele-ophthalmology platforms can provide 24/7 guidance, clarify postoperative instructions, and reduce clinician message loads [23-25]. For example, Ittarat et al. [23] demonstrated how conversational agents could reinforce self-management behaviors for chronic eye conditions (e.g., glaucoma, diabetic retinopathy). These systems can dynamically tailor explanations by health-literacy level, language, and age, fostering culturally sensitive education [26].

Ophthalmology is particularly suited to combining visual and textual information. For example, LLMs can enhance understanding by providing plain-language explanations alongside annotated images, such as labeled fundus photographs or schematic diagrams of the eye. Combined with retrieval-augmented generation (RAG), these assistants can ground their responses in trusted ophthalmic literature, ensuring both clarity and factual accuracy [14,53].

These systems can tailor complex information to diverse health-literacy levels by improving patients' ability to understand and apply health information-two of the six domains in the widely recognized health-literacy framework [23]. Asynchronous LLM assistants can reinforce postoperative counseling, support informed consent, and facilitate chronic-disease self-management [24]. Embedding LLM chatbots within portals can help address common questions (e.g., medication effects, scheduling), reducing message burden on staff [25]. Adaptive content generation by age, language, and prior knowledge can promote culturally sensitive, scalable patient education [26].

Diagnostic performance benchmarks

Ophthalmology Board-Style Examinations 

Having outlined major application domains, we next summarize how LLMs perform on formal ophthalmic benchmarks. Multiple studies comparing GPT iterations with legacy models and humans show progressive improvements in ophthalmic knowledge and reasoning [27].On ophthalmology board-style multiple-choice questions, newer GPT models have outperformed GPT-3.5 and equaled or surpassed humans in several datasets [28]. These gaps underscore rapid gains in domain-specific fluency, reasoning, and comprehension with more advanced LLMs [29].

Such advancements support educational use cases, including the generation of practice questions and feedback aligned with subspecialty content [30]. LLM performance varies across subspecialties and task types, with consistently lower scores on image-dependent questions versus text-only items [31]. Recent evaluations confirm that multimodal GPT variants (e.g., GPT-4o, GPT-4V) improve on image-based tasks compared with text-only LLMs, though challenges remain [32].

Across independent exam-style datasets, LLM performance shows a clear step-up from GPT-3.5 to GPT-4 and contemporary peers, often approaching or matching mean human scores. Studies using OKAP-like and board-style questions consistently report that GPT-4 outperforms GPT-3.5 and aligns with, or exceeds, typical human examinee accuracy, while Gemini/Copilot performance is mixed and dataset-dependent [2,5]. Importantly, gains reflect not just factual recall but improvements in chain-of-thought diagnostic reasoning on ophthalmic stems, including distractor rejection and guideline-consistent management selections [2,5].

To help readers compare results across papers, Table 1 summarizes model versions, dataset types, domains, and headline outcomes, and highlights methodological caveats (e.g., image exclusion, question leakage controls, and rubric strictness).

**Table 1:** Diagnostic performance of large language models (LLMs) on ophthalmology board-style and case-based benchmarks

Study Title	Model(s) Evaluated	Question Source/Dataset	Subspecialty Focus	Image Use	Primary Metric (Accuracy/Score)	Human Comparator/Baseline	Key Findings and Limitations
*Performance of Generative Large Language Models on Ophthalmology Board-Style Questions* – Cai et al., 2023	GPT-4 vs. GPT-3.5	OKAP-style MCQs (≈250 items)	General + Neuro-ophthalmology	No	GPT-4 82% vs. GPT-3.5 69%	Mean human ≈ 76%	GPT-4 matched or exceeded resident-level performance; text-only format limits external validity [2].
*Comparison of GPT-3.5, GPT-4, and Humans on Ophthalmology Exams* – Lin et al., 2023	GPT-3.5, GPT-4, Humans	Exam-style Q-bank items	Mixed subspecialties	No	GPT-4 ≈ 78%, Humans ≈ 76%	Resident mean	GPT-4 excelled on text-based items but underperformed on image-dependent questions [26].
*Accuracy of Large Language Models in Answering Ophthalmology Board-Style Questions: A Meta-Analysis* – Wu et al., 2024	GPT-3.5, GPT-4, Gemini	Aggregated board datasets > 4 studies	General, Retina, Cornea	Mixed	Weighted mean ≈ 80% (95% CI 77–83%)	Human range: 74–82%	Confirmed consistent GPT-4 superiority; variability due to dataset composition [5].
*Evaluating the Performance of ChatGPT on Board-Style Examination Questions in Ophthalmology: A Meta-Analysis* – Wei et al., 2025	ChatGPT (GPT-4 API)	1,000 Board-style MCQs	General Ophthalmology	No	81 ± 2%	N/A	High factual accuracy and internal consistency; lacked image-based tasks [30].
*Assessing the Performance of Microsoft Copilot, GPT-4 and Google Gemini in Ophthalmology* – Silhadi et al., 2025	GPT-4, Gemini, Copilot	Cornea & Retina cases (≤ 25% image)	Cornea, Retina	Mixed	GPT-4 84%, Gemini 79%, Copilot 73%	Expert review	GPT-4 strongest clinical reasoning; Gemini more concise but occasionally incomplete [31].
*Multimodal Performance of GPT-4 in Complex Ophthalmology Cases* – Mikhail et al., 2025	GPT-4 (text + image)	Complex case reports (30 scenarios)	Mixed/Advanced diagnostics	Yes	73% correct diagnoses	Expert panel 81%	GPT-4 approached expert accuracy but hallucinated rationales in ≈ 20% of cases [7].
*Performance of GPT-4V (Vision) in Ophthalmology* – medRxiv 2024 Preprint	GPT-4V (Vision variant)	Fundus + Optical Coherence Tomography (OCT) benchmarks	Retina, Glaucoma	Yes (> 90%)	75–78% (AUROC ≈ 0.82)	Expert annotators	Multimodal input improved diagnostic precision; errors in fine lesion detection [32].
*Toward Expert-Level Medical Question Answering with Large Language Models* – Singhal et al., 2025	Med-PaLM 2, GPT-4	Multispecialty QA set (Ophth subset)	Cross-disciplinary	No	Med-PaLM 2 ≈ 85% on Ophth subset	N/A	Domain-tuned LLMs highest factuality; curated training reduced hallucinations [27].
*Assessment of a Large Language Model’s Responses to Questions and Cases About Glaucoma and Retina Management* – Huang et al., 2024	GPT-4	Glaucoma & Retina cases	Glaucoma, Retina	Mixed	72–80%	Attending review	Correct answers often paired with flawed rationales; supports dual grading scheme [54].

Across multiple datasets and study designs, successive GPT iterations consistently improved on ophthalmology items relative to earlier models and often approached or matched mean human performance. Head-to-head studies report that GPT-4 outperforms GPT-3.5 on ophthalmology board-style questions, with meta-analyses confirming an aggregate advantage for newer models and narrowing the gap with human comparators [2,5,26,30]. These gains extend beyond raw accuracy; several investigations note better answer justification quality, improved handling of multi-step reasoning, and more consistent selection among closely related distractors compared with legacy LLMs [2,26,30]. However, inter-study heterogeneity remains substantial due to differing question banks, item difficulty distributions, and whether explanations were scored alongside final answers [5,26,30].

Importantly, benchmark validity depends on item provenance and leakage control. Few studies can exclude overlap between test questions and public text corpora used for pretraining; this raises the possibility that part of the apparent advantage reflects memorization rather than reasoning. Variability in prompt style, temperature, and chain-of-thought display further complicates cross-study comparisons, emphasizing the need for standardized evaluation protocols for ophthalmology question sets [5,26,30].

Performance is systematically higher on text-only items than on image-dependent questions across multiple evaluations [31,32]. Recent work with multimodal GPT variants (e.g., GPT-4V/4o) shows incremental gains when ophthalmic images accompany the vignette. Yet, explanations may still contain visual-reasoning errors even when the final choice is correct, underscoring an emerging "right answer, wrong rationale" risk profile [7,32,43]. These patterns suggest that benchmarks should report disaggregated outcomes by item type (text-only vs. image-based), visual modality (fundus, OCT, slit-lamp), and required reasoning step (recognition vs. synthesis vs. management) [7,31,32,43].

Reported strengths are greatest in domains where narrative clues dominate, such as general and neuro-ophthalmology, with GPT-4 approaching the level of board-certified specialists in case-based neuro-ophthalmic scenarios [4,9,33]. By contrast, accuracy tends to dip in image-centric subspecialties; studies in corneal disease and retina/glaucoma management demonstrate that LLMs benefit from multimodal inputs but still lag human subspecialists on nuanced grading and stage-specific management choices [31,36,54]. In primary-care-like triage of common eye complaints, LLMs can reach clinically useful performance and generate safe initial advice, though escalation thresholds vary by model and prompt design [8,10]. Collectively, these results argue for subspecialty-stratified reporting tables and for pairing LLMs with structured image features when used for differentiating look-alike entities [4,8-10,31,33,36].

Comparative studies indicate that GPT-4 generally outperforms GPT-3.5 and is competitive with, or superior to, other contemporary systems (e.g., Gemini, Copilot) on mixed ophthalmology datasets, although advantages are not universal and can reverse in specific domains or item types [26,31]. When images are included, multimodal GPT variants improve over text-only baselines but still produce occasional hallucinated visual rationales [7,32,43]. Exam-bank-based evaluations and OKAP-style head-to-heads corroborate these patterns, with between-model spreads narrower on foundational knowledge and wider on subspecialty reasoning and management sequencing [2,26,31,49].

Multimodal Systems and Retrieval-Augmented/Domain-Specific Models

Multimodal AI systems that combine language with imaging inputs can enhance diagnostic accuracy by leveraging complementary data streams [33]. For example, adding ophthalmic figures to GPT-4 changes task performance profiles and highlights the impact of visual context on diagnostic outputs [34]. Purpose-built ophthalmic multimodal benchmarks further show gains for retina, cornea/external disease, and glaucoma when images are incorporated [35]. Explainable outputs (e.g., heatmaps, textual rationales) can support clinician trust and streamline interpretation workflows [36]. These approaches are already being explored for pediatric retinal disease assessment, glaucoma staging, and diabetic retinopathy screening [37]. As multimodal systems strengthen EHR and imaging integration, they could accelerate comprehensive, case-level assistance in routine care [38].

Emerging multimodal architectures such as GPT-4V(ision), GPT-4o, and ophthalmology-specific variants integrate textual reasoning with visual feature extraction [7,32,33]. Studies that append ophthalmic images to identical text stems show significant accuracy gains, particularly in distinguishing mimicking pathologies such as central vs. branch retinal vein occlusion or optic neuritis vs. compressive neuropathy [7,33,36]. Improvements are most pronounced in retina, cornea/external disease, and glaucoma, though fine-grade findings, such as microaneurysm density or early Retinal Nerve Fiber Layer (RNFL) loss, remain challenging [36]. Explainability modules (heatmaps, textual rationales) enhance clinician trust but cannot fully prevent hallucinations; several reports describe correct answers accompanied by flawed reasoning [43,44]. Consequently, human-in-the-loop validation remains mandatory.

RAG and ophthalmology-tuned LLMs demonstrate improved factual grounding and citation behavior by constraining outputs to curated corpora (guidelines, peer-reviewed articles, and institutional protocols). An ophthalmology-specific RAG framework reduced unsupported assertions and improved evidence linkage in diagnostic explanations, demonstrating that point-of-care accuracy can be improved without internet access to broad, noisy sources [14]. Early in-domain models (e.g., EYE-Llama) aim to encode specialty vocabulary and task structure; while still preliminary, they represent a complementary path to reliability alongside general-purpose systems. Simulated history-taking and "digital patient" evaluations also show that structured ophthalmic knowledge injected via scenarios can enhance signal fidelity and trainee skill acquisition while offering measurable endpoints for LLM evaluation [38].

Enhancing LLM Accuracy Through Retrieval and Specialization Models 

More recently developed versions of LLMs use domain-specific fine-tuning and RAG to meet the requirements of privacy compliance and information accuracy [39]. Models such as ChatZOC and EYE-Llama are trained on carefully selected ophthalmology texts, clinical guidelines, and peer-reviewed case studies rather than on general internet databases. These models can retrieve documents that are relevant to the input when proposing diagnoses [40]. This helps guarantee that the provided information is based on reliable sources that represent the most up-to-date clinical perspective [41]. Retrieval-augmented LLMs provide highly contextualized diagnostic support, increase factual correctness, and dramatically lower the danger of hallucinations. Hospitals and clinics can use these models in a manner to fit their own record systems, including their patient data, imaging archives, pathology reports, etc. This enables hospitals and clinics to have systems that tailor to each patient's personalized predictive analytics, automated documentation, and personal clinical guidelines [42].

Discussion

LLMs have rapidly evolved into valuable tools across multiple domains of ophthalmology. Recent studies demonstrate that GPT-4 and comparable models approach expert-level diagnostic reasoning, achieving accuracies above 80% on board-style and case-based ophthalmic assessments, significantly outperforming earlier versions such as GPT-3.5 [2,5,26,27,30]. Beyond diagnostic applications, LLMs have shown measurable benefits in workflow efficiency, reducing clinical documentation time by up to 40% and improving chart quality through ambient scribing and automated EHR integration [11-17]. In parallel, these systems enhance patient communication and medical education, generating materials with superior readability and accessibility compared to conventional sources [21-23,52-54]. The introduction of multimodal and retrieval-augmented models, including GPT-4V and EYE-Llama, marks a shift toward integrating text and imaging data to deliver more contextually grounded and clinically relevant information [7,14,32,53]. Collectively, these findings underscore the growing translational relevance of LLMs in ophthalmology, highlighting their potential to augment diagnostic reasoning, streamline documentation, and expand access to high-quality ophthalmic education and care.

When stratified by subspecialty, general ophthalmology and neuro-ophthalmology show the highest diagnostic concordance (≈ 85-90%) due to their reliance on structured narratives [4,7]. In contrast, retina, cornea/external disease, and glaucoma remain more challenging because these tasks depend on image-based pattern recognition (e.g., microaneurysm density, keratic precipitates, cup-to-disc asymmetry) [5,7,31,36]. Multimodal variants such as GPT-4V and GPT-4o modestly improve visual-text reasoning but do not yet reach expert parity [7,32,33]. Performance also declines as case complexity increases, particularly for rare or multi-step conditions where models generate partially correct or incomplete rationales [43,44]. Furthermore, benchmark heterogeneity significantly influences results. Some studies employ single-turn standardized prompts, while others allow multi-step or "chain-of-thought" reasoning that can inflate accuracy by 5-10% [5,26,27,30]. Question sources also differ; some reuse public board questions, risking information leakage, whereas de novo or blinded question sets yield more conservative but realistic results [27,30,31]. These disparities highlight the need for uniform benchmarking protocols that incorporate prompt limits, consistent scoring rubrics, and the inclusion of multimodal items. Model lineage and data curation further explain variation. General-purpose LLMs (GPT-3.5, Gemini, Copilot) are trained on broad internet text and may misinterpret ophthalmic terminology such as "cup-to-disc ratio" or "Seidel test." In contrast, domain-tuned or retrieval-augmented models (ChatZOC, EYE-Llama) integrate ophthalmology-specific guidelines, clinical reports, and peer-reviewed literature, yielding higher factual accuracy and fewer hallucinations [14,53]. Their ability to retrieve and cite verified ophthalmic sources enhances both factual grounding and interpretability, underscoring the benefit of specialization over scale alone.

The clinical relevance of these systems is threefold. First, diagnostic and triage support: LLMs like GPT-4 can assist ophthalmologists in differentiating urgent from routine presentations, enhancing tele-ophthalmology triage in low-resource environments [8,10,23]. Second, workflow optimization: the adoption of ambient LLM scribes and automated note generation has been shown to improve efficiency [11-13,17,19]. This is particularly valuable in ophthalmology, where documentation is detailed and repetitive. Third, patient education: conversational LLMs improve clarity and accessibility of medical information, surpassing traditional handouts in readability and engagement [21,22]. For clinicians, these improvements mean less administrative burden, better-informed patients, and more equitable access to ophthalmic expertise. When incorporated into EHR systems, LLMs can also cross-reference past visits, imaging reports, and pathology data to create personalized clinical summaries [15,16,42]. As multimodal integration becomes routine, these systems may evolve into decision-support tools capable of recommending guideline-consistent management plans for common ocular diseases.

Limitations and Challenges

Despite progress, hallucinations (fluent but inaccurate statements) remain a central risk in clinical use of LLMs [43]. Reported error patterns in multimodal variants show accurate final choices can still be paired with flawed rationales, especially for image tasks [44]. Because ophthalmic diagnosis often hinges on precise image interpretation and nuanced context, hallucinations can jeopardize patient safety [45]. Accordingly, LLM outputs should complement, not replace, clinician judgment and be subject to expert review before action [46].

Visual-language models may obtain correct classifications with incorrect or fabricated visual rationales, a failure mode with safety implications for education and clinical support [43-50]. Additional concerns include dataset leakage, lack of consensus prompt standards, and unreported temperature/decoding settings that impede replication. Rare-disease coverage and subspecialty edge cases remain underrepresented in benchmarks, aligning with broader evidence that general LLMs struggle with uncommon pathologies and multimodal signals that extend beyond text and 2D images [50]. Future benchmarks should preregister protocols, release item-level metadata (modality, difficulty, cognitive step), and include calibration and explanation-quality scoring, not just top-one accuracy [5,31,32,43,50].

In fields where multimodal data, such as histology, genetic markers, or electrophysiology, are important for diagnosis, these models often lack a strong understanding of the field, making it difficult to use them in practice [50,51]. This highlights the importance of combining various multimodal data sets while adjusting their knowledge of subspecialities and constantly checking their accuracy [52]. Most clinical illnesses rarely follow written norms and often involve multiple conditions or an unusual disease presentation. This discrepancy, therefore, poses a risk for real-world implementation.

This review also has inherent limitations. As a structured narrative review, it does not follow a fully systematic methodology, and the search strategy may have missed relevant articles that were not indexed in the databases queried or were published outside the selected time frame. Only English-language publications were included, which may introduce language bias. In addition, rapid developments in both general-purpose and ophthalmology-specific LLMs mean that some recent or ongoing studies may not have been captured at the time of writing. Finally, while the review synthesizes key themes across clinical, technical, and ethical domains, it does not formally assess study quality or risk of bias and should therefore be interpreted as a descriptive overview rather than a quantitative meta-analysis.

Ethical, Legal, and Regulatory Considerations

Key concerns include accountability for AI-generated content, consent and transparency, and protection of personally identifiable information [47]. Privacy-preserving designs and governance (e.g., data minimization, logging, human-in-the-loop reviews) are necessary for responsible deployment [48]. Ethical frameworks emphasize clear disclosure, bias monitoring, and equitable access when integrating LLMs into three of six ophthalmic care [49]. With extensive generalized training, LLMs have been able to gain a strong understanding of ophthalmologic knowledge and reasoning. However, when presented with rare illnesses or subspecialty-specific pathologies, diagnostic accuracy and efficiency decrease significantly [50]. These models struggle to even identify rare diseases, yet they are excellent at assessing presentations of commonly seen ophthalmic diseases.

Educational Utility

LLMs have become reliable educational tools for medical students preparing for board exams [52]. These systems have been able to both pass board-style exams and provide students with interactive learning tools, such as practice questions. Multimodal systems such as EyeTeacher and EyeAssistant use personalized GPT models to generate evidence-based clinical questions, simulate exam questions, and provide clinical explanations [52]. Not only can they be used as online tutors for medical students and residents, but they can also serve as an educational resource to keep physicians up to date with current research [53].

With the ability to compile recent research, simplify complex concepts, and provide continuous feedback, LLMs have become intelligent and adaptive learning systems that can align with what their users already know [54-56]. These models have enabled students in remote areas or settings with limited resources to receive an excellent ophthalmic education [52-54]. By increasing access to information and closing educational disparities, these AI systems have contributed to the development of training settings that are more equal on a global scale [51-54]. Future LLMs could include virtual reality settings that allow for an immersive training experience in pathology recognition, clinical examination simulations, and surgical experience.

Future Directions

The future of LLMs will be determined by several significant advances and policy developments in the domains of clinical viability, reliability, and ethics. A first step toward implementing LLMs in clinical settings is to further develop and use multimodal AI systems that can analyze and interpret both visual and textual information. With this as an initial starting point, the diagnostic and therapeutic reach of LLMs will be greatly expanded. If these multimodal systems can interpret and analyze OCT scans, fundus photographs, visual data, research literature, and patient histories in one, this would be a revolutionary step towards AI-assisted ophthalmic care.

A second step for the widespread use of AI systems in clinical care is implementing a framework that addresses data management and patient confidentiality. These frameworks should support the training of AI models on decentralized datasets, meaning data remains stored at local institutions rather than being pooled into a central repository. This approach helps maintain patient privacy by minimizing data transfer and exposure. In addition to privacy, these frameworks must incorporate rigorous testing protocols to ensure the models meet clinical performance thresholds, address edge-case scenarios, and operate within clearly defined decision-making boundaries.

Furthermore, well-designed frameworks would enable secure, ethical data sharing across international healthcare institutions, thereby promoting global collaboration among clinicians. By providing access to ethically sourced and demographically diverse datasets, such systems could help reduce model bias and improve generalizability while ensuring compliance with local and international data protection regulations. In addition to ensuring compliance with HIPAA and clinical requirements, devoting time to refining domain-specific models and clinician-AI collaboration platforms will be crucial.

Ultimately, if these systems are able to be context-aware, follow ethical guidelines, and have outstanding performances in ophthalmic care while maintaining a structured yet flexible clinical protocol, then the widespread use of LLMs in clinical care will be a large part of medicine in the future.

## Conclusions

A fundamental change in the way ophthalmologic care will be practiced and delivered is represented by LLMs. Diagnostics, education, and workflow optimization can benefit greatly from their capacity to analyze and understand natural language, extract clinically important information, and engage in human-like conversations with users. By implementing controlled administrative procedures, integrating LLMs can improve clinical documentation, democratize patient contact, customize health education, and improve diagnostic accuracy through guided reasoning. An era of intelligent, fair, and effective medicine can be achieved by carefully integrating LLMs into clinical care, with appropriate precautions, collaborative oversight, and a patient-centered design ethic.
